# Treatment of Preschool Children With Autism Spectrum Disorder: A Trial to Evaluate a Learning Style Profile Intervention Program in China

**DOI:** 10.3389/fped.2022.831621

**Published:** 2022-03-16

**Authors:** Chen-huan Ma, Ling-yan Chen, Meng-fan Li, Dan Wu, Sha-sha Wang, Yan-jun Zhao, Jian-li Shi, Patrick J. Rydell, Jin-jin Chen, Yu Wang

**Affiliations:** ^1^Department of Child Health Care, Shanghai Children’s Hospital, Shanghai Jiao Tong University, Shanghai, China; ^2^Department of Molecular, Cellular and Developmental Biology, University of Colorado, Denver, CO, United States; ^3^Department of Communicative Disorders, University of Louisiana at Lafayette, Lafayette, LA, United States

**Keywords:** autism spectrum disorder (ASD), learning style profile (LSP), social behavior, ASD symptom severity, early intervention

## Abstract

**Objective:**

To investigate whether the provision of learning style profile (LSP) training improves development in preschool children with autism spectrum disorder (ASD) in China and to describe the characteristics of children who benefit from the intervention.

**Methods:**

Eighty-one children aged 36 to 72 months who were diagnosed with ASD for the first time were recruited for the intervention group. All of them received 24 weeks of LSP training, consisting of hospital- and home-based training. Twenty-one children with ASD of the same age in the control group had never received any intervention after diagnosis but underwent an assessment. Assessments were conducted at baseline and 24 weeks later. Differences in the developmental level and severity of ASD symptoms over time and between groups were analyzed by repeated standardized measures. Secondary analyses examined age effects among the 36– 48-, 48– 60-, and 60–72-month age groups.

**Results:**

Within-group comparison of the intervention group revealed significant treatment effects after the intervention, according to: language, social and adaptive developmental quotients (DQs) of the China Developmental Scale; total Childhood Autism Rating Scale (CARS) score; and hyperactivity, peer problems, total difficulties, and prosocial behavior scores of the Strengths and Difficulties Questionnaire (SDQ). Similar gains were observed in gross and fine motor DQs of the China Developmental Scale and emotional symptoms and conduct problems scores of the SDQ; however, the differences between these pre- and postintervention scores did not reach statistical significance. Comparisons among the three age groups in the intervention groups demonstrated a significant age effect on adaptive DQs of the China Developmental Scale; total CARS score; hyperactivity, peer problems and total difficulties scores of the SDQ. Comparison between the intervention and control groups revealed significant treatment effects on language, social and adaptive DQs of the China Developmental Scale; total CARS score; and emotional symptoms, conduct problems, hyperactivity, peer problems, total difficulties, and prosocial behavior scores of the SDQ after the intervention. Similar gains were observed in gross and fine motor DQs of the China Developmental Scale, although differences between the two groups did not reach statistical significance.

**Conclusion:**

Our findings suggest that LSP training can effectively improve social behavior and reduce the severity of ASD symptoms in children with ASD. Our data also highlight the importance of early intervention.

## Introduction

Autism spectrum disorder (ASD) is a set of heterogeneous neurodevelopmental conditions characterized by persistent impairment in reciprocal social communication and social interaction, as well as stereotypical patterns of behavior, interests, or activities that are present from early childhood and limit or impair everyday functioning (1). The prevalence of ASD has been steadily increasing since the first epidemiological study of ASD, and the most recently reported estimate was approximately 1 in 44 children (2). This rise has resulted in increasing social and financial burdens. Although the pathogenesis of ASD is generally thought to arise from interactions among genetic and environmental factors, a specific mechanism has yet to been identified (3). While ASD has been considered a severe and chronic disability that persists for decades, the most effective interventions have been behavioral and educational (4). Many studies have shown that early intervention is beneficial to both the short-term and long-term outcomes of children with ASD (5–7).

Evidence-based strategies to treat children with ASD were first developed in the 1980s, when Lovaas reported the results of a controlled study showing that an intervention based on applied behavior analysis (ABA) could significantly increase intellectual and educational functioning in children with ASD (8). That study caused a paradigm shift regarding treatment efficacy due to the significant improvements and even “recovery” for almost half of the children with ASD after receiving excellent treatment with early intervention at a sufficient intensity. Models based on ABA originating from the Lovaas method are collectively referred to as early intensive behavioral intervention (EIBI) (9), which involves manual-based intensive programs and targets a comprehensive series of skills for training, practice, and generalization (10). Improvements afforded by EIBI, which is recognized as a preferred intervention for children with ASD, have been demonstrated in the areas of adaptive behavior, IQ, communication, socialization, and daily living skills (11–13). While EIBI is effective, some children who participate in EIBI fail to generalize newly developed skills across more circumstances, exhibit challenging escape/avoidance behaviors, or show lack of spontaneity and overdependence on prompts (14).

Although traditional ABA intervention models remain widely used today, naturalistic developmental models have been created to incorporate ABA-based principles into a developmental framework. Naturalistic developmental behavioral interventions (NDBIs) are receiving increased attention due to their consistency with the characteristics of children’s learning processes (14). NDBIs involve shared control between the child and therapist in natural settings, and emphasize play, social interaction, and communication initiation by the child, which allows the embedding of many learning opportunities and natural consequences (15). An increasing number of studies have demonstrated the effects of NDBIs, including focused and comprehensive interventions (5, 16, 17).

One such developmental intervention is learning style profile (LSP) training. As an emerging intervention for ASD, LSP integrates the core challenges of ASD and well-established assessment and intervention guidelines from existing ASD intervention models. All children have their own strategies or preferences that could help them acquire information from the environment around them to establish social interactions, which can be viewed as learning styles; however, for children with ASD, differences in learning style may severely limit their ability to notice others in the environment around them, which will further contribute to their deficit in engaging in and learning from social interactions. Due to learning style challenges, these children may subsequently experience difficulties in developing shared meanings, shared affect, shared emotions and, eventually, conventional behaviors (18). The atypical learning style of children with ASD leads to interaction challenges in social situations. Instead of teaching children with ASD all they need to know, LSP intervention attempts to describe a variety of patterns, strategies and preferences to show how children with ASD may be learning and acquiring information from the environment around them (18).

Patrick J. Rydell, the author and developer of LSP, selected program components that represent the greatest challenges for families and teachers, based on the learning style differences of children with ASD to help them independently learn how and when to use skills they have already acquired without direction and prompting from other people. Furthermore, it is fully recognized that different children with ASD have different learning style. Consequently, LSP training attempts to profile these learning style differences for the purpose of matching specific intervention methods to each individual child with ASD. LSP can be interpreted as a tree consisting of roots, a trunk and a crown, representing emotional regulation, joint attention and the ten LSP components, respectively. The ten LSP components are Object vs. People Orientation; Learns through Social Modeling, Demonstration and Rehearsal; Attains Social Cues from Multiple Partners; Level of Flexibility with Objects, Activities and People; Shared Control; Interaction Style; Verbal/Symbolic Communication; Executive Function; Distance Learning; and Transitions (18). Children with ASD can learn well and establish joint attention and social interaction only when they are in a good emotional state. Family support also needs to be emphasized in the LSP approach, as it is crucial for Chinese children with ASD. Currently, although rehabilitation modalities have been gradually developed and demonstrated some achievements, there remains a dearth of adequate intervention systems in the field of ASD rehabilitation in China. Simultaneously, providing intervention guidance following diagnosis is a significant challenge for clinicians. In addition, hospital-based and rehabilitation center-based interventions are influenced by geographical location and rehabilitation costs. For the reasons presented above, parent-based interventions have become more important and been proven effective by a growing number of studies (19, 20).

Although there has been little research examining the effectiveness of LSP intervention which is a simplified and practical version of the Social Communication, Emotional Regulation, and Transactional Support (SCERTS) model, the SCERTS model has been proven effective for children with ASD (21, 22). Given the need in China, we introduced LSP training and assessed its effects on the developmental level and ASD symptom severity in Chinese children with ASD, to expand the application of LSP training in China.

## Materials and Methods

### Participants

The participants were Chinese children aged 36–72 months who were enrolled in the Department of Child Health Care of Shanghai Children’s Hospital with a diagnosis of ASD from July 2018 to December 2019. The inclusion and exclusion criteria of the intervention group are described in detail below. The inclusion criteria were as follows: (i) a senior developmental and behavioral pediatrician and a senior psychiatrist made diagnosis of ASD based on the criteria listed in the Diagnostic and Statistical Manual of Mental Disorders, 5th edition (DSM 5) (1); (ii) the children were aged 36–72 months; (iii) the children had never received any training; and (iv) the parents or caregivers of the children needed to understand all the content of the study and sign the informed consent form to allow their child to participate in the research following a consultation with the researchers during the enrollment. The exclusion criteria were as follows: all children were eligible for inclusion except those with a brain injury; fragile X syndrome or other syndromes resulting from known genetic defects or inherited metabolic diseases; and other diagnosed conditions involving impairments in social or communication abilities, such as intellectual disability without ASD, schizophrenia, language disorders and social communication disorder. Children were both required to meet the criteria described above and to receive 24 weeks of LSP training (18, 22). For children with ASD, it usually takes around 24 weeks to establish social communication and conversational foundations in the LSP intervention (18). Patrick J. Rydell, the author and developer of LSP, makes assessments for children with ASD once 24 weeks in his center. If a participant interrupted the training three or more times during the study, they were also excluded.

For children who are first diagnosed with ASD, we recommend intervention as early as possible; however, some children cannot receive timely intervention because of parental perception of ASD, accessibility of rehabilitation centers or for other reasons. Therefore, we selected children who could not receive intervention at Shanghai Children’s Hospital as controls. The inclusion and exclusion criteria of the control group were the same as those for the intervention group, except for participation in the intervention. These children did not receive any training for 24 weeks after diagnosis, but did undergo assessment at Shanghai Children’s Hospital. If the participants received the intervention during the 24 weeks, they were excluded.

In the intervention group, 90 participants were included at the beginning of the study, but 6 could not continue to participate in the study because of their geographical location, and 3 participants received other interventions at the same time. In the control group, 61 participants were included at the beginning, but 33 were excluded because they received interventions.

### Assessment Procedure

Standardized assessment tools were used before the intervention. Twenty-four weeks following the first assessment, the children were reassessed using the same measures. Assessments were conducted at Shanghai Children’s Hospital by clinicians and parents for 2 h. A single-blind study design was used, and to ensure blinding, the clinicians were not aware of whether the child had received the intervention. In addition, it was important to ensure that the same clinician and parent completed the pre- and postintervention assessments.

### Measures

#### Developmental Level

The China Developmental Scale for Children is a clinician-rated observation instrument that measures a child’s gross motor, fine motor, adaptive, language and social functioning, according to the developmental level of children in China; it has shown high reliability and validity (23, 24). The evaluation indices are the mental age and developmental quotient (DQ) (mental age = the sum of scores for the 5 scales/5, DQ = mental age/actual age × 100). The reference ranges of the DQ are as follows: > 130, excellent; 110–129, good; 80–109, medium; 70–79, critically low; and <70, a mental developmental disorder (25).

#### Severity of Autism Spectrum Disorder Symptoms

The severity of ASD symptoms was assessed using the Childhood Autism Rating Scale (CARS) and the Strengths and Difficulties Questionnaire (SDQ). The CARS is a clinician-rated questionnaire based on observations of children with ASD, as well as information from parents and/or teachers, with accepted sensitivity (26). The CARS includes the following 15 dimensions: Relating to People, Imitation, Emotional Response, Body Use, Object Use, Adaptation to Change, Visual Response, Listening Response, Taste, Smell and Touch Response and Use, Fear or Nervousness, Verbal Communication, Non-verbal Communication, Activity Level, Level and Consistency of Intellectual Response, and General Impressions (27). These dimensions are scored on a four-point scale to assess autism severity, where 1 is normal, 2 is mildly abnormal, 3 is moderately abnormal and 4 is severely abnormal; total scores range from 15 to 60. The suggested cutoff points were used to categorize ASD into three categories: no ASD (15–30), mild/moderate ASD (31–36), and severe ASD (37–60) (27).

The SDQ is a brief behavioral screening questionnaire completed by parents/caregivers of children that has been used worldwide. It consists of 25 items across five subscales, including Emotional Symptoms, Conduct Problems, Hyperactivity, Peer Problems, and Prosocial Behavior. All these scales, except prosocial behaviors, are added together to generate a total difficulty score. The score of each subscale ranges from 0 to 10, with higher scores indicating more problems, except for the prosocial behavior subscale, for which a lower score indicates more problems. The SDQ has been introduced and formally adapted to the Chinese language with accepted reliability and validity (28, 29). Cutoff scores are recommended to identify a child with high risk of behavioral problems (emotional symptoms > 4, conduct problems > 3, hyperactivity problems > 7, peer problems > 5, prosocial behaviors <5, and total difficulties > 16) (28).

### Interventions

Participants in the intervention group were enrolled in a research-based 24-week comprehensive intervention using LSP training, including hospital- and home-based training at Shanghai Children’s Hospital.

#### Hospital-Based Training

Before the start of the training, the therapists worked with parents to identify their relevant concerns and formulated an individualized program for each child according to the child’s strengths and weaknesses in learning style. In identifying the characteristics of the child’s learning style and designing relevant intervention programs, questions related to the ten LSP components were asked. Each LSP component was assessed on an arrow divided into four degrees representing the continuous development of a child’s learning style: does not know at all, knows a little bit, has started using, and uses habitually (Supplementary Figure 1). Each ability within the LSP component changes from less to more, and the child achieves a balanced progression of abilities. The learning style characteristics of each child were positioned according to the degree of the LSP components that best described him or her to develop an individualized LSP intervention program.

The creation of an individualized program required not only selection of the characteristics among the ten LSP components that best represented the learning style of the child in the natural environment but also consideration of the goals that would conform to the child’s needs, development, and program focus—according to which intervention priorities for each LSP component were modified and adapted. For example, for LSP 1, Object vs. People Orientation, it was asked whether the child with ASD primarily focused on objects or on a social partner in the process of social learning. Many cues in the environment can help a child understand how to participate and communicate; however, if children keep their heads down, indicating that they are not paying attention or learning in relation to people, they will miss much information. In this situation, there could be consideration of introducing objects by demonstrating, modeling and rehearsing an activity in which both the partners and objects are equally important, for the purpose of establishing joint attention and a “we” learning style orientation (18).

The therapists provided hospital-based training for each child, according to the previously developed individualized LSP intervention program. For this training, the child needed to be present at the hospital for 1 h per week. Parents could participate in the entire training to enable them to carry out training at home by themselves.

#### Home-Based Training

Home-based training was performed at the child’s home at a convenient time for the families with the aim of providing continuity and promoting the generalization of skills across the child’s environments. Parents learned to carry out training at home and using relevant training videos provided by the therapists. If parents had any questions or encountered any problems during the training, they could contact the therapists for help. In addition, the therapists spent 15 min communicating with parents about home-based training and providing guidance before every session of hospital-based training. The home-based training required 3 h daily. To monitor the implementation of the intervention by parents, telephone follow-up was conducted weekly, and outpatient follow-up was conducted monthly.

### Data Analysis

Pre- and postintervention differences were assessed as the changes in each outcome variable. T tests were conducted to compare the participants in terms of developmental level and ASD symptom severity. For exploratory analysis stratified by age, participants were divided into three age groups: 36–48, 48–60, and 60–72 months. Correlation analysis was performed for comparisons among age groups. A significance level of *P* < 0.05 was considered statistically significant.

## Results

### Demographics

The main demographic characteristics of the children, parents and families in the intervention and control groups are presented in Table 1. Of the 81 participants in the intervention group, 33 were in the 36–48-month age group [Group 1 (G1)], 25 were in the 48–60-month age group [Group 2 (G2)] and 23 were in the 60–72-month age group [Group 3 (G3)]. Of the 28 participants in the control group, 21 were in the 36–48-month age group [Group 1c (G1c)], 5 were in the 48–60-month age group [Group 2c (G2c)] and 2 were in the 60–72-month age group [Group 3c (G3c)]. Due to the significant difference in the number of participants between G2 and G2c and between G3 and G3c, between-group comparisons were not possible; therefore, only G1c was included in the data analysis. No significant differences were found between G1 and G1c or among age groups with respect to sex, residence, family income, father’s education, mother’s education or ASD symptom severity (χ^2^ = 1.074, 3.038, 4.501, 0.955, 0.851, 9.306, *P* > 0.05; χ^2^′ = /, 0.128, 1.211, 1.496, 2.568, 0.790, *P* > 0.05, respectively).

**TABLE 1:** Demographic characteristics of the intervention and control groups.

		Intervention group			Control group				
Category		G1	G2	G3	G1c	χ^2^	*P* value	χ^2′^	*P*’ value
Age	/	33	25	23	21	/	/	0.183	0.856
Sex	Boys	25	20	20	17	1.074	0.585	/	0.747
	Girls	8	5	3	4				
Residence	Shanghai	22	21	19	13	3.038	0.219	0.128	0.721
	Nearby Shanghai	11	4	4	8				
Family income	<200,000 yuan	7	6	3	4				
	200,000–500,000 yuan	8	11	10	8	4.501	0.342	1.211	0.546
	≥500,000 yuan	18	8	10	9				
Father’s Education	High school degree or below	5	4	5	1				
	Bachelor’s degree	15	12	9	13	0.955	0.917	1.496	0.473
	Master’s degree or above	13	9	9	7				
Mother’s Education	High school degree or below	8	5	7	2				
	Bachelor’s degree	14	11	8	13	0.851	0.931	2.568	0.277
	Master’s degree or above	11	9	8	6				
Severity of ASD symptoms	Mild	10	2	4	7				
	Moderate	15	21	14	11	9.306	0.054	0.790	0.674
	Severe	8	2	5	3				

*G1, age 36–48-month in the intervention group; G2, age 48–60-month in the intervention group; G3, age 60–72-month in the intervention group; G1c, age 36–48-month in the control group.*

### Outcome Measurements

Changes in all outcomes from pre-intervention [Time 1 (T1) to post-intervention and Time 2 (T2) were assessed]. Further, age stratification was conducted to compare the outcomes among age groups.

#### Assessment of Developmental Level

Within-group comparisons of the intervention group revealed significant increases from pre- to post-intervention in language, social, and adaptive DQs of the China Developmental Scale for Children (*t* = 5.03, 2.92, *P* < 0.01; *t* = 2.08, *P* < 0.05), while no significant increases were detected in gross and fine motor DQs (*t* = 1.03, 0.00, *P* > 0.05) (Table 2). In comparisons of the three age groups in the intervention group, adaptive DQs of children in G1 increased more significantly (*r* = –0.28, *P* < 0.05) than those of participants in the other age groups (Table 2). Comparison of G1 and G1c showed no significant differences in gross motor, fine motor, language, social and adaptive DQs of the China Developmental Scale for Children at T1 (*t* = 0.61, 1.25, 0.00, 7.52, 1.39, *P* > 0.05); however, language, social and adaptive DQs of this scale were significantly higher in G1 than G1c at T2 (*t* = 4.89, 5.74, 6.15, *P* < 0.01). Similar gains were observed in gross and fine motor DQs, but the differences between the two groups did not reach statistical significance (*t* = 0.67, 0.00, *P* > 0.05) (Table 3).

**TABLE 2:** Comparison of T1 and T2 scores on the China Developmental Scale for Children in the intervention group.

Item	Group	T1	T2	*t*	*P* value	*r*	*P*’ value
Gross	G0	84.79 (11.11)	85.56 (6.42)	1.03	0.306	–	–
motor	G1	81.48 (12.03)	82.88 (7.11)	1.22	0.231		
	G2	88.92 (10.46)	88.72 (5.75)	0.16	0.878	–0.12	0.302
	G3	85.04 (9.13)	85.96 (4.21)	0.60	0.554		
Fine	G0	82.48 (10.34)	82.48 (6.63)	0.00	1.000	–	–
motor	G1	79.03 (11.91)	80.18 (8.08)	1.24	0.223		
	G2	85.44 (9.95)	84.92 (5.47)	0.45	0.658	–0.17	0.122
	G3	84.22 (6.59)	83.13 (4.13)	1.422	0.169		
Language	G0	66.21 (9.32)	68.35 (8.66)	5.03	< 0.001*	–	–
	G1	64.97 (9.74)	66.82 (8.16)	3.23	0.003*		
	G2	69.20 (9.40)	71.04 (8.07)	3.35	0.003*	0.06	0.627
	G3	64.74 (8.17)	67.61 (9.61)	2.59	0.017**		
Social	G0	68.90 (10.88)	71.02 (8.21)	2.92	0.005*	–	–
	G1	66.30 (11.42)	68.36 (8.38)	2.05	0.048**		
	G2	71.16 (9.83)	74.88 (5.98)	2.09	0.048**	–0.02	0.878
	G3	70.17 (10.86)	70.65 (8.75)	0.60	0.556		
Adaptive	G0	71.86 (11.48)	73.11 (8.1)	2.08	0.040**	–	–
	G1	69.79 (12.22)	71.91 (8.12)	2.09	0.044**	–0.28	0.012*
	G2	72.24 (10.68)	74.40 (8.67)	2.12	0.044**		
	G3	74.43 (11.11)	73.43 (7.75)	1.06	0.303		

T1, pre-intervention; T2, post-intervention. G0, intervention group; G1, 36–48-month age group; G2, 48–60-month age group; G3, 60–72-month age group. Data expressed are the means (SD). *P < 0.01, **P < 0.05.

**TABLE 3:** Comparison of T1 and T2 scores on the China Developmental Scale for Children between G1 and G1c.

Item	T	G1	G1c	*t*	*P* value
Gross motor	T1	81.48 (12.03)	80.17 (9.64)	0.61	0.549
	T2	82.88 (7.11)	81.35 (8.71)	0.67	0.527
Fine motor	T1	79.03 (11.91)	80.24 (10.05)	1.25	0.222
	T2	80.18 (8.08)	80.31 (9.62)	0.00	1.021
Language	T1	64.97 (9.74)	64.71 (9.26)	0.00	1.018
	T2	66.82 (8.16)	64.92 (9.08)	4.89	<0.001*
Social	T1	66.30 (11.42)	65.72 (10.76)	7.52	0.157
	T2	68.36 (8.38)	65.81 (11.04)	5.74	<0.001*
Adaptive	T1	69.79 (12.22)	68.72 (8.94)	1.39	0.173
	T2	71.91 (8.12)	68.45 (9.61)	6.15	<0.001*

T1, pre-intervention; T2, post-intervention. G1, age 36–48-month in the intervention group; G1c, age 36–48-month in the control group. Data are expressed as the means (SD). *P < 0.01.

#### Assessment of Autism Spectrum Disorder Symptom Severity

Within-group comparison of the intervention group showed that total CARS score was significantly decreased at T2 (*t* = 9.00, *P* < 0.01) (Table 4). Further, comparison among the three age groups of the intervention groups revealed that children in G1 showed a more obvious change in this outcome than those in the other two groups (*r* = −0.029, *P* < 0.05) (Table 4). Comparison of G1 and G1c revealed no significant difference in total CARS score T1 (*t* = 1.33, *P* > 0.05), with a significantly lower score in G1 at T2 (*t* = 10.64, *P* < 0.01) (Table 5).

**TABLE 4:** Comparison of T1 and T2 CARS scores in the intervention group.

Group	T1	T2	*t*	*P* value	*r*	*P*’ value
G0	35.36 (5.15)	32.79 (4.53)	9.00	<0.001*	–	–
G1	34.70 (5.07)	31.42 (3.57)	5.96	<0.001*		
G2	34.76 (5.23)	32.52 (5.15)	7.05	<0.001*	–0.029	0.040**
G3	36.96 (4.70)	35.04 (4.35)	3.93	0.001*		

CARS, Childhood Autism Rating Scale. T1, pre-intervention; T2, post-intervention. G0, intervention group; G1, 36–48-month age group; G2, 48–60-month age group; G3, 60–72-month age group. Data are expressed as the means (SD). *P < 0.01, **P < 0.05.

**TABLE 5:** Comparison of T1 and T2 CARS scores between G1 and G1c.

T	G1	G1c	*t*	*P* value
T1	34.70 (5.07)	35.08 (5.76)	1.33	0.221
T2	31.42 (3.57)	35.17 (6.02)	10.64	<0.001*

CARS, Childhood Autism Rating Scale. T1, pre-intervention; T2, post-intervention. G1, age 36–48-month in the intervention group; G1c, age 36–48-month in the control group. Data are expressed as the means (SD). *P < 0.01.

Within-group comparisons of the intervention group revealed significant reductions in hyperactivity, peer problems and total difficulties scores, and increases in prosocial behavior score of the SDQ (*t* = 6.91, 7.29, 7.78, 6.57, *P* < 0.01), while there were no significant decreases in emotional symptoms and conduct problems scores (*t* = 1.97, 0.35, *P* > 0.05) (Table 6). Comparison of the three age groups in the intervention group showed more obvious decreases in hyperactivity and peer problems scores in G1 (*r* = −0.29, −0.26, *P* < 0.05), while total difficulties score decreased less obviously in G3 (*r* = −0.35, *P* < 0.01) than those of the other age groups (Table 6). Comparison of G1 and G1c demonstrated no significant differences in emotional symptoms, conduct problems, hyperactivity, peer problems, total difficulties or prosocial behavior scores of the SDQ at T1 (*t* = 1.98, 0.98, 1.83, 1.01, 0.26, 0.17, *P* > 0.05). Significant reductions were observed in G1 at T2 in conduct problems, emotional symptoms, hyperactivity, peer problems, and total difficulties scores as well as an increase in prosocial behavior score on the SDQ (*t* = 2.38, *P* < 0.05; *t* = 5.76, 5.88, 6.04, 8.79, 7.01, P < 0.01) (Table 7).

**TABLE 6:** Comparison of T1 and T2 SDQ scores of the intervention group.

Item	Group	T1	T2	*t*	*P* value	*r*	*P*’ value
Emotional	G0	8.43 (1.38)	8.22 (1.53)	1.97	0.052	−	−
symptoms	G1	8.30 (1.45)	8.06 (1.54)	1.25	0.222		
	G2	8.84 (0.62)	8.72 (1.21)	0.62	0.543	0.28	0.805
	G3	8.17 (1.78)	7.91 (1.73)	1.82	0.083		
Conduct	G0	4.58 (1.49)	4.54 (1.41)	0.35	0.731	−	−
problems	G1	4.36 (1.66)	4.15 (1.40)	1.49	0.147		
	G2	4.80 (1.38)	4.64 (1.25)	0.89	0.382	–0.20	0.077
	G3	4.65 (1.34)	5.00 (1.48)	1.45	0.162		
Hyperactivity	G0	8.48 (1.31)	7.62 (1.66)	6.91	< 0.001*	−	−
	G1	8.30 (1.26)	7.00 (1.64)	5.82	< 0.001*		
	G2	9.04 (0.94)	8.56 (1.29)	2.39	0.025**	–0.29	0.010**
	G3	8.13 (1.58)	7.48 (1.65)	4.04	0.001*		
Peer	G0	8.37 (1.43)	7.65 (1.55)	7.29	< 0.001*	−	−
problems	G1	9.09 (0.77)	8.12 (1.24)	5.66	< 0.001*		
	G2	8.16 (1.03)	7.52 (1.23)	4.23	< 0.001*	–0.26	0.019**
	G3	7.57 (1.97)	7.13 (2.05)	2.647	0.015**		
Prosocial	G0	2.00 (1.43)	2.65 (1.34)	6.57	< 0.001*	−	−
behavior	G1	1.82 (1.74)	2.61 (1.71)	4.30	< 0.001*		
	G2	2.08 (0.95)	2.84 (0.80)	4.88	< 0.001*	–0.16	0.159
	G3	2.17 (1.40)	2.52 (1.24)	2.34	0.029**		
Total	G0	29.86 (4.69)	28.04 (4.44)	7.78	< 0.001*	−	−
difficulties	G1	30.06 (4.70)	27.33 (4.37)	6.91	< 0.001*		
score	G2	30.84 (3.18)	29.44 (3.28)	4.29	< 0.001*	–0.35	0.001*
	G3	28.52 (5.81)	27.52 (5.38)	2.48	0.021**		

SDQ, strengths and difficulties questionnaire; T1, pre-intervention; T2, post-intervention; G0, intervention group; G1, 36–48-month age group; G2, 48–60-month age group; G3, 60–72-month age group. Data are expressed as the means (SD). *P < 0.01, **P < 0.05.

**TABLE 7:** Comparison of T1 and T2 SDQ scores between G1 and G1c.

Item	T	G1	G1c	t	P value
Emotional symptoms	T1	8.30 (1.45)	8.74 (1.53)	1.98	0.051
	T2	8.06 (1.54)	9.38 (2.17)	5.76	< 0.001*
Conduct problems	T1	4.36 (1.66)	4.64 (1.71)	0.98	0.325
	T2	4.15 (1.40)	4.71 (1.38)	2.38	0.026**
Hyperactivity	T1	8.30 (1.6)	8.21 (1.55)	1.83	0.082
	T2	7.00 (1.64)	8.37 (2.04)	5.88	< 0.001*
Peer problems	T1	9.09 (0.77)	9.14 (2.02)	1.01	0.237
	T2	8.12 (1.24)	9.30 (1.93)	6.04	< 0.001*
Prosocial behavior	T1	1.82 (1.74)	1.91 (1.25)	0.26	0.839
	T2	2.61 (1.71)	1.88 (1.11)	7.01	< 0.001*
Total difficulties score	T1	30.06 (4.70)	30.19 (5.72)	0.17	0.996
	T2	27.33 (4.37)	30.75 (5.19)	8.79	< 0.001*

SDQ, Strengths and Difficulties Questionnaire. T1, pre-intervention; T2, post-intervention. G1, age 36–48-month in the intervention group; G1c, age 36–48-month in the control group. Data are expressed as the means (SD). *P < 0.01, **P < 0.05.

## Discussion

This study was the first trial conducted to evaluate LSP intervention program in China. The primary aim of this study was to evaluate whether, after 24 weeks of treatment, Chinese preschool children with ASD showed significant improvement in their developmental level and ASD symptom severity across a variety of measures, both observed and reported. The outcomes of children aged 36–72 months enrolled in hospital- and home-based LSP training were examined. In our study, between the preintervention assessment and 24-week follow-up assessment, there were significant improvements in social and language DQs of the China Developmental Scale for Children, as well as significant reductions in hyperactivity score and peer problems score and improvement in prosocial behavior score of the SDQ; these findings suggest that the participants achieved significant improvements in their social communication skills. The significant gains in adaptive DQ indicate that the participants may have shown a reduction in stereotyped behavior patterns. In addition, the intervention was also successful in reducing the severity of ASD symptoms, as shown by CARS scores.

Deficits in social communication and stereotyped behavior patterns are the core symptoms of ASD. The LSP intervention aims to establish social communication and conversational foundations for children with ASD by focusing more on the child’s relative strengths and weaknesses related to learning style and less on percentages of correctness of skills. For example, a child with ASD may primarily learn in relation to objects but be less oriented toward people, which results in missing information from social cues, models and related demonstrations. Contrary to interventions focused on object-oriented, task completion-oriented interventions, with relatively few learning opportunities provided to establish joint attention with partners for social communication, the LSP intervention aims to establish joint attention and a “we” learning style orientation between the child and partner. The partner introduces objects by demonstrating, modeling and rehearsing an activity so that the child with ASD begins to learn ways of interacting with the object based on a “people-oriented” learning style (18). After a social communication foundation has been established, pragmatic social objectives can be embedded in the program in a step-by-step manner.

Engaging as an active participant is a prerequisite for optimal children learning. Further, the skills that are easiest for children to learn are those that are just beyond their present knowledge (30). Therefore, assessing the current degree of ability of children with ASD and choosing adjacent development areas as goals can help to achieve success. This approach helps children to connect new experiences with their existing knowledge and enables them to discover patterns in the world around them through systematical increases in the complexity of learning experiences. Children’s initiative and spontaneity are fostered and rewarded by such interventions, which further promotes their contributions to their own learning (14). In this study, we identified the characteristics of each child’s learning style that were best positioned in each degree of the LSP components, to develop an individualized LSP intervention program.

Our results are consistent with reports demonstrating that children with ASD improve in several areas when they are involved in NDBI intervention programs, targets of which generally include the entire range of developmental domains, covering cognition, social, language, and play (13). Dawson et al. (5) demonstrated the efficacy of the Early Start Denver Model (ESDM) in improving cognitive and adaptive behavior and reducing ASD symptom severity of toddlers with ASD. Landa et al. (31) reported that an early achievement model could improve socially engaged imitation in toddlers with ASD, while Morgan et al. (21) showed that the SCERTS model could improve active engagement, adaptive communication, social skills, and executive functioning in children with ASD.

This study found no statistically significant difference in motor DQ in the China Developmental Scale for Children or the scores for emotional symptoms or conduct problems in the SDQ. Early sensory and motor differences and possible emotional regulation differences are a prodrome of ASD that manifests in the latter half of the first year of life before the appearance of social communication and restrictive behavioral differences that are more directly related to ASD diagnostic criteria (32, 33). Parsons et al. (34) showed no significant changes in the visual motor skills of children with ASD after they received an early intervention with a tablet-based information communication technology application. We believe that the focus of LSP training on addressing the core challenges of ASD highlighted in the literature contributed to the present results. In addition, we note that the LSP training conducted in this study lasted for 24 weeks and that it will be necessary and valuable to continue to assess changes in the motor and emotional symptoms, and behavioral problems of children with ASD.

Age effects were also observed for adaptive DQs of the China Developmental Scale, total CARS score and hyperactivity, peer problems, and total difficulties scores of the SDQ. Our awareness of the early behavior and education of children with ASD has increased in recent years. In establishing an ASD diagnosis, medical/genetic counseling, medical management, family support, educational interventions, and guidance for appropriate intervention programs for the child should be provided. Several studies have also reported similar results. Vivanti et al. (35) found that younger children achieved greater verbal DQ gains than their older counterparts after receiving ESDM for 1 year. Jansson et al. (36) reported that children with ASD aged 2.5 years showed wide variability in adaptive and global functioning outcomes after receiving an ABA intervention.

### Limitations and Future Directions

Although this study was implemented using a rigorous approach, we note some limitations. Clearly, the level of evidence of the present study is lower than that of a randomized controlled trial. Consequently, a randomized controlled trial to further examine the effects of LSP intervention on Chinese children with ASD is under development. Additionally, without knowing how well the parents delivered the LSP training at home, we cannot guarantee the quality and consistency of the home-based training, although some measures were taken. Future directions for this research will include providing more guidance for home-based LSP training online and offline. Notably, the involvement of parents in the intervention may have positively affected their perceptions of their child’s behaviors. Thus, as a parent-rated scale, the SDQ may be subject to the effects of parental involvement in the intervention. In further studies, rating scales and observation instruments completed by professionals who are not directly involved in the intervention need to be used to evaluate and compare changes in children’s behaviors as objectively as possible. Finally, the Autism Diagnostic Observation Schedule (ADOS) is widely adopted in studies on patients with ASD worldwide; however, we started to use the ADOS after recruitment of participants. In further studies on ASD, the ADOS for evaluation of children with ASD will be added.

## Conclusion

Treatment of children with ASD presents several challenges. There is a lack of evidence regarding the application of LSP intervention in the Chinese context. This study is the first to evaluate the effectiveness of LSP training in treating preschool children with ASD in China, contributing to the limited body of research on LSP training in this country. We demonstrate that LSP training is feasible and has a significant impact on Chinese preschool children with ASD. Furthermore, we emphasize the necessity and importance of early intervention.

## Data Availability Statement

The original contributions presented in the study are included in the article/Supplementary Material, further inquiries can be directed to the corresponding authors.

## Ethics Statement

The studies involving human participants were reviewed and approved by Medical Research Ethics Committee of Shanghai Children’s Hospital, China. Written informed consent to participate in this study was provided by the participants’ legal guardian/next of kin.

## Author Contributions

J-JC, YW, and C-HM designed the study. M-FL and S-SW formulated the treatment program. PR and J-LS provided treatment guidance. M-FL, S-SW, and DW provided training and consultation. L-YC performed the assessments. Y-JZ performed the statistical analysis. C-HM and YW completed the first version of the manuscript. All authors critically revised the final version of the manuscript.

## Conflict of Interest

The authors declare that the research was conducted in the absence of any commercial or financial relationships that could be construed as a potential conflict of interest.

## Publisher’s Note

All claims expressed in this article are solely those of the authors and do not necessarily represent those of their affiliated organizations, or those of the publisher, the editors and the reviewers. Any product that may be evaluated in this article, or claim that may be made by its manufacturer, is not guaranteed or endorsed by the publisher.

## Supplementary Material

Supplementary Figure 1 Continuous development of a child’s learning styles in each LSP component.
