# Supplementary figures and images for: Treatment of Preschool Children With Autism Spectrum Disorder: A Trial to Evaluate a Learning Style Profile Intervention Program in China
Source: Front Pediatr. 2022 Mar 16;10:831621. doi: 10.3389/fped.2022.831621 (PMC8966376; doi:10.3389/fped.2022.831621)

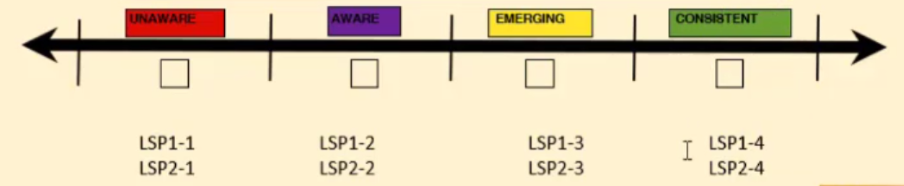

Supplement: Supplementary Figure 1 — Continuous development of a child’s learning styles in each LSP component. [file Image_1.PNG]
